# CoFormerNet: A Transformer-Based Fusion Approach for Enhanced Vehicle-Infrastructure Cooperative Perception

**DOI:** 10.3390/s24134101

**Published:** 2024-06-24

**Authors:** Bin Li, Yanan Zhao, Huachun Tan

**Affiliations:** 1 School of Transportation, Southeast University, Nanjing 211189, China; lbin@outlook.com; 2 School of Mechanical Engineering, Beijing Institute of Technology, Beijing 100081, China; zyn@bit.edu.cn; 3 Department of Transportation Engineering, Beijing Institute of Technology, Zhuhai 519088, China; 4 ShenSi Lab, Shenzhen Institute for Advanced Study, University of Electronic Science and Technology of China, Shenzhen 518110, China

**Keywords:** V2X, cooperative perception, 3D LiDAR object detection

## Abstract

Vehicle–infrastructure cooperative perception is becoming increasingly crucial for autonomous driving systems and involves leveraging infrastructure’s broader spatial perspective and computational resources. This paper introduces CoFormerNet, which is a novel framework for improving cooperative perception. CoFormerNet employs a consistent structure for both vehicle and infrastructure branches, integrating the temporal aggregation module and spatial-modulated cross-attention to fuse intermediate features at two distinct stages. This design effectively handles communication delays and spatial misalignment. Experimental results using the DAIR-V2X and V2XSet datasets demonstrated that CoFormerNet significantly outperformed the existing methods, achieving state-of-the-art performance in 3D object detection.

## 1. Introduction

In autonomous driving systems, establishing an accurate representation of the driving environment is crucial. This not only concerns the safe operation of vehicles but also the safety of passengers and the surrounding environment [1]. Autonomous vehicles mainly rely on onboard LiDAR sensors to capture dense point clouds of the surrounding environment and perform object detection. The system must be capable of promptly detecting and classifying all significant objects on the path, including blind spots, obstructions, and distant objects. Failure of the perception system could result in serious accidents, as demonstrated by incidents involving autonomous vehicles from Tesla [2,3] and Uber [4]. In these accidents, factors such as blind spots, obstructions, and long-distance perception played significant roles [5]. To address these challenges, researchers proposed the concept of cooperative perception [6], which involves integrating information from infrastructure-side sensors to mitigate these limitations. Due to the elevated installation height of infrastructure-side sensors, autonomous vehicles can achieve a global perspective and long-distance perception by receiving information from infrastructure-side sensors, significantly enhancing their perception capabilities. This approach is more reliable and cost-effective compared with single-point perception. A report [7] indicates that vehicle–infrastructure cooperative perception greatly improves the safety of autonomous driving, reducing takeover events by 62% and lowering single-vehicle costs by 30%.

**Figure 1:**
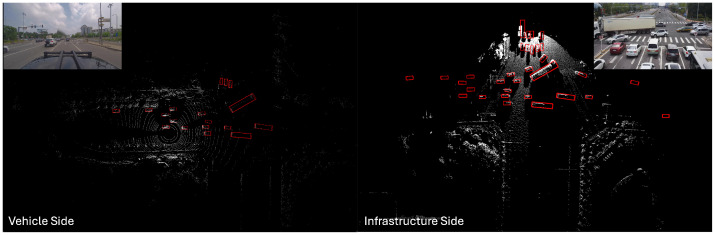
Perception conditions under vehicle–infrastructure cooperation in typical intersection traffic scenarios. The red box indicates the detected objects.

In traffic scenarios, particularly at intersections and T-junctions, blind spots, obstructions, and distant obstacles that are missed can create numerous potential safety issues. Sensors mounted on elevated infrastructure can effectively address these problems. In terms of obstacle detection, LiDAR is widely used for 3D object detection; estimating the size, 3D posture, and category of objects in the environment; and assigning them to 3D bounding boxes. As shown in Figure 1, the LiDAR point cloud from both the vehicle and infrastructure sides at the same moment reveals partial misdetections caused by blind spots and obstructions. Additionally, the greater distance results in fewer point clouds, leading to significant misdetections and false detections. The goal of detecting objects outside the field of view or that are obscured is to enhance the perception capabilities of the vehicle’s autonomous driving system in vehicle–infrastructure cooperative scenarios, thereby improving traffic safety through better planning of its future trajectory.

Three-dimensional object detection is a key function in vehicle–infrastructure cooperative perception systems, with LiDARs and cameras extensively used for this task. The advantages and disadvantages of each were widely compared in many studies [6,8,9,10]. In vehicle–infrastructure systems, both the vehicle and infrastructure are typically equipped with both LiDARs and cameras. However, multimodal fusion often occurs on a single side, as in these studies focused on multimodal sensor fusion for individual vehicles, with the same algorithms applicable to the infrastructure side. The fusion of sensors at both the vehicle and infrastructure ends generally happens at a single-sensor-type level, and LiDAR-based fusion tends to perform significantly better than camera-based fusion. Therefore, continuing the research direction of previous studies, this article focuses mainly on LiDAR-based fusion for 3D detection in vehicle–infrastructure cooperation.

In the realm of fusion work, the primary methods include early fusion, late fusion, and middle fusion. Early fusion involves transmitting raw data directly, late fusion involves merging information at the object level, and middle fusion involves integrating intermediate features for feature fusion. In practical applications, due to computational delays and the transmission channel bandwidth, the data received at the vehicle end, regardless of the fusion method employed, represents information from a past and uncertain time point. In recent years, a considerable number of algorithms have neglected the impact of these delays, focusing instead on designing superior fusion techniques, which is impractical for real-world applications. Recently, some research has started to address and attempt to resolve issues related to these delays, though there is still limited focus on fusion methods themselves. In this study, we took both aspects into account and proposed a novel cooperative detection framework called CoFormerNet. Instead of attempting to reduce these delays or predict features to match the vehicle end’s timestamp, we shifted the focus to more effectively utilizing and integrating historical features to enhance the precision of vehicle-end perception. First, we used a fundamentally consistent network architecture on the vehicle and infrastructure sides, and simultaneously fused intermediate features at two different stages. The first stage of intermediate feature fusion occurs after extracting the bird’s eye view (BEV) features. The infrastructure-side uses the temporal aggregation module (TAM) to aggregate the historical BEV features. Due to the inevitability of the communication delay, the vehicle-side TAM treats the BEV features aggregated by the infrastructure-side as historical features for fusion, effectively avoiding the problems brought about by the communication delay. The second stage of intermediate fusion is in the decoding layer. To alleviate the vehicle–infrastructure sensor calibration problem caused by the hard association strategy, we used a cross-attention mechanism to construct a soft association between the vehicle and the infrastructure LiDAR. The vehicle-side uses spatial-modulated cross-attention (SMCA) to softly link the already fused BEV features of the vehicle side with the object queries obtained by the infrastructure side, enabling the network to adaptively determine where and what information should be taken from the fused BEV features of the vehicle side.

The main contributions of this work are as follows:

- CoFormerNet uses the TAM to fuse the historical temporal information of the infrastructure sensors, which not only fully utilizes the computational capabilities of the infrastructure but also fully exploits the advantage that the long-distance global perspective of the infrastructure can extend the perception field of the vehicle in space and time. At the same time, intermediate feature fusion at two different stages, fully utilizing infrastructure perception information, solves the impact of time-asynchronous uncertainty due to communication delay and the calibration bias of vehicle–infrastructure sensors on feature fusion.
- The design of CoFormerNet allows for end-to-end training, thereby fully integrating vehicle–infrastructure perception information. This design requires only one model to cover all possible communication delays, greatly reducing the complexity of the model. This end-to-end training method can better optimize the model’s performance, improving its application effects in real environments.
- CoFormerNet achieved a new state-of-the-art level using the DAIR validation set. On the DAIR validation set, CoFormerNet’s performance showed a significant improvement over the previous best results. This indicates that the design and methods of CoFormerNet have significant advantages in enhancing the perception capabilities of autonomous driving systems and are expected to promote the further development of autonomous driving technology.

The design goal of CoFormerNet is to address the aforementioned problems in a simple and unified manner. Through this approach, we hope to enhance the perception capabilities of autonomous driving systems, thereby improving their safety and performance in complex traffic environments. The implementation of this method may have a profound impact on the future of autonomous driving, propelling it toward higher safety and efficiency.

## 2. Related Work

### 2.1. Egocentric Perception

The field of 3D object detection using LiDAR point cloud data can be categorized into point-based, grid-based, and transformer-based methods. Point-based methods operate directly on the sparse and unordered set of points in the LiDAR point cloud to predict 3D bounding boxes. These methods aggregate point features through multiscale/multiresolution grouping and set abstraction techniques. Examples of point-based methods include PointRCNN [11], PVRCNN [12], and Frustum-PointNet [13]. Although these methods can achieve large receptive fields, they tend to be computationally expensive. Grid-based methods address the sparsity and unordered nature of LiDAR point clouds by projecting the points onto regular grids, such as voxels [14,15], BEV pillars [16], or range projection [17,18]. They use 3D convolutional neural networks (CNNs) to extract voxel-wise features. PIXOR [19] and PointPillars [16] are examples of grid-based methods. These methods are less computationally expensive compared with point-based methods but may result in a loss of 3D information. Transformer-based methods have also been explored in 3D object detection. DETR [20] is a transformer architecture that formulates the 2D detection problem as direct set prediction, removing the need for non-maximum suppression (NMS). Some methods use transformers for the feature extraction network, such as Voxel Transformer [21], Pointformer [22], and CWT [23]. 3DETR [24] is the first end-to-end transformer model used for 3D object detection. This model can directly predict the category, position, and size of 3D objects from point cloud data. VISTA [25] enhances 3D object detection by fusing multiview features from different perspectives using a dual cross-view spatial attention mechanism. BoxR [26] introduced a novel attention mechanism called Box-Attention, which is used in 2D and 3D transformer models. Box-Attention enables the transformer model to better learn spatial information, thereby improving its performance in various visual tasks. Li3DeTr [27] can directly predict the category, position, and size of 3D objects from the LiDAR point cloud data. This work is the first to apply the deformable attention architecture to LiDAR-based 3D object detection models.

The high cost of using LiDAR systems has made them unsuitable for large-scale deployment. An alternative solution emphasizes cost-effectiveness, attempting to use more economical sensors, such as relying solely on cameras for real-time 3D object detection [28,29,30,31]. However, in most cases, 3D detection using only cameras significantly underperforms compared with LiDAR-based detection [10]. Therefore, using cameras for 3D detection is significantly less reliable in terms of safety compared with LiDAR-based systems. Some approaches combine LiDAR with cameras for 3D detection, where the camera primarily serves a supportive role. Yet, this integration increases the computational demands due to the addition of more data sources. At the same time, as the usage of LiDAR increases, its cost is rapidly decreasing, reaching a stage where it can be economically scaled up.

### 2.2. Vehicle–Infrastructure Cooperative Perception

The objective of V2X perception is to detect objects in traffic environments using sensors on vehicles and other devices, which involves the problem of multi-agent sensor fusion. Depending on the fusion stage, V2X perception can be categorized into early, intermediate, and late fusion types [6].

Early fusion directly transforms and merges raw data, forming comprehensive perception. For example, in [32], the authors propose combining point clouds from different perception points to enhance 3D object detection. This involves transmitting each point cloud to a central fusion system, then connecting them into a single point cloud and inputting it into the detection model. Another study [33] converted point cloud data into depth information at the pixel level, and then connected it to RGB images. This method can effectively handle data from different sensors and improve the accuracy of object detection. Ref. [34] applied sparse convolution to support detection in low-density point cloud data. This method can effectively handle large amounts of sensor data and improve the perception efficiency.

Late fusion is a practical technique used in the cooperative perception of V2X, where perception results from different agents are combined. For example, [35,36] discussed how to merge separate sensor outputs at the decision level to create the final perception. This method can effectively handle data from different sensors and improve the accuracy of object detection. In [37], the authors propose a multi-sensor fusion method for handling data from different sensors. They used a deep learning model to fuse data from radar, LiDAR, and cameras to enhance the object detection accuracy. The advantages of late fusion include the ability to integrate information from different models without the need for additional training and providing the flexibility to integrate the results from different data modalities. However, late fusion can also be challenging due to the need for the precise alignment of each output, and the complexity of the result fusion process may increase. Additionally, late fusion has the disadvantage of error accumulation.

Intermediate fusion is the main scheme in cooperative perception model design, where the perception model of the self-driving vehicle integrates its own intermediate features with the intermediate features of other cooperative agent models, achieving cooperative perception. For example, in [38], the authors propose a deep fusion scheme to merge regional features from multiple views. This allows for interaction between the intermediate layers of different paths. This method can effectively handle data from different sensors and improve the accuracy of the object detection. Ref. [39] proposes a spatial perception information transmission mechanism for joint inference detection and prediction. This method can better understand the objects in the environment and their motion by transmitting information in space. Ref. [40] proposes a communication framework to avoid unnecessary transmission between connected vehicles, thereby reducing the communication bandwidth. This method can effectively handle large amounts of sensor data and improve the perception efficiency. In [41], the authors considered the impact of the information transmission delay on perception. They proposed a method that can effectively perform data fusion under a limited communication bandwidth and transmission delay. A novel flow-based intermediate feature fusion module is proposed in FFNet [42], which effectively combines features from vehicles and infrastructure, introducing a more accurate motion modeling approach to capture the dynamic changes of objects. These examples highlight the efficacy of intermediate fusion in handling multi-sensor data and its potential to enhance the object detection accuracy.

## 3. Method

**Figure 2:**
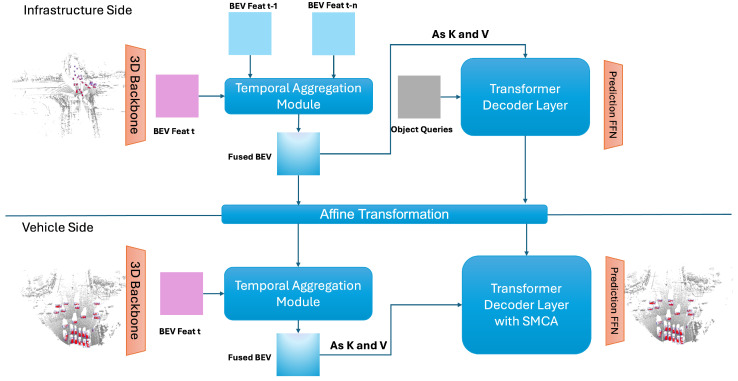
CoFormerNet overview.

In this work, we propose a new transformer-based framework to address the problem of vehicle–infrastructure cooperative 3D object detection. As shown in Figure 2, CoFormerNet uses a fundamentally consistent architecture in both the vehicle and the infrastructure branches. The structure of each branch is similar to that of the LiDAR branch in [8]. After BEV feature extraction on the infrastructure side, we used the temporal aggregation module to fuse historical BEV features. At the same time, we fused the intermediate features of the vehicle and infrastructure sides in terms of temporal and spatial features at two stages within this process. In Section 3.1, we explain how the temporal aggregation module is used in the fusion of infrastructure-side historical BEV features and the fusion with vehicle-side BEV features. In Section 3.2, we discuss how the spatial information on both the vehicle and infrastructure sides is fused, addressing the impact on the final accuracy due to calibration bias. In Section 3.3, we briefly describe the affine transformation of infrastructure features and their processing and compression during transmission. Finally, in Section 3.4, we explain the CoFormerNet training method, including a description of the end-to-end learning framework.

### 3.1. Temporal Features Fusion Stage

Temporal information is crucial for perception systems to understand their surroundings. Without temporal clues, inferring the speed of moving objects from static images or detecting highly occluded objects is a challenging task. On the other hand, due to the broad field of view on the infrastructure side, infrastructure features that have integrated historical temporal information can greatly enhance the perception capabilities of the vehicle side. For the infrastructure branch, the sensors are fixed and the viewpoint does not change; therefore, the historical features and current features are always aligned. The challenge we need to address is how to accurately associate the same objects between BEV features at different times.

CoFormerNet adopts a temporal aggregation module (TAM) to fuse historical BEV features. Specifically, in a recurrent way, we first used a spatial attention layer similar to the convolutional block attention module (CBAM) [43] to calculate pixel-level attention based on current BEV features. We then connected the current BEV features and the previously weighted BEV features. Next, the system adaptively learned the receptive field through a deformable attention mechanism, enabling it to effectively capture significant features in the aligned BEV features. Finally, we obtained temporally fused BEV features through additional convolutional layers.

**Figure 3:**
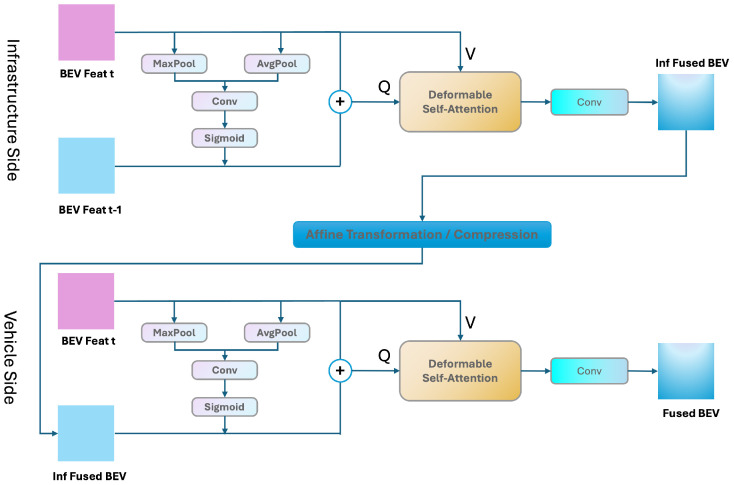
Temporal aggregation module.

Figure 3 illustrates the detailed structure of our temporal aggregation module (TAM). Let us denote the BEV features at times *t* and t−1 on the infrastructure side as $B_{\inf}^{t}$ and $B_{\inf}^{t-1}$, respectively. Then, the feature $B_{\inf}^{t-1,t}$, which is the result of the infrastructure fusing historical BEV features, can be represented as

(1) $$\begin{array}{cc} B_{\inf}^{t-1,t} & =\mathrm{TAM}(B_{\inf}^{t-1},B_{\inf}^{t})\\ \; & =\mathrm{Conv}(\mathrm{DeformAttn}\left(\tilde{B}\inf^{t-1,t},B\inf^{t}\right)), \end{array}$$

where TAM represents the temporal aggregation module fusion process, Conv represents a convolution operation, the deformable attention mechanism is calculated by DeformAttn, and ${\tilde{B}}_{\inf}^{t-1,t}$ is the result after fusion through the CBAM. The process can be formulated as follows:

(2) $$\begin{array}{c} {\tilde{B}}_{\inf}^{t-1,t}=\mathrm{Concat}\left(B_{\inf}^{t},\mathrm{Mul}\left(F\left(B_{\inf}^{t}\right),B_{\inf}^{t-1}\right)\right), \end{array}$$

where the function *F* can be formulated as follows:

(3) $$\begin{array}{c} F\left(B_{\inf}^{t}\right)=\sigma \left(\mathrm{Conv}\left(\left[\mathrm{AvgPool}\left(B_{\inf}^{t}\right),\mathrm{MaxPool}\left(B_{\inf}^{t}\right)\right]\right)\right), \end{array}$$

where σ denotes the sigmoid function.

Before the fusion of the vehicle–infrastructure BEV features, due to the differences in geographical location and direction between vehicle and infrastructure sensors, the BEV feature map fused on the infrastructure side needs to be transformed into the vehicle’s coordinate system for feature fusion. Moreover, to reduce the amount of transmission, the features need to be further compressed. The specific process is explained in detail in Section 3.2. Here, we use ${\hat{B}}_{inf}^{t-1,t}$ to represent the historical fusion features of the infrastructure side of the BEV after undergoing a transformation.

At the same time, there is an unavoidable communication delay between the vehicle and the infrastructure. This delay is usually random and uncertain. Directly combining vehicle-side BEV features with time-fused infrastructure-side BEV features can lead to serious feature mismatch problems. Following the same mechanism, we treated time-fused infrastructure-side BEV features as historical features, transforming the communication delay problem into a historical time information fusion problem. We used the same temporal aggregation module to model this temporal connection between the vehicle–infrastructure BEV features. This process can be formulated as follows:

(4) $$\begin{array}{cc} B_{veh}^{fused} & =TAM({\hat{B}}_{inf}^{t-1,t},B_{veh}^{t}) \end{array}$$

where $B_{veh}^{fused}$ denotes the final vehicle-side fused BEV features. The subsequent process is similar to the above mentioned infrastructure-side fusion. Since our history fusion model only needs the final BEV feature of the previous frame, it can easily be deployed for online prediction by saving BEV features in a memory bank.

### 3.2. Feature Transformation, Compression, and Decompression

Due to the geographical location and orientation differences between the vehicle and infrastructure sides, the feature map fused by the infrastructure side needs to be transformed into the vehicle’s coordinate system for feature fusion. Affine transformation [44] can be used for this purpose. The affine transformation is a specific category of projection transformation that does not move any object from affine space to the plane at infinity and vice versa. To achieve this, the infrastructure sends the fused feature map ${\hat{B}}_{inf}$ containing historical information and pose information to the vehicle side. The pose information represents the position and direction, represented as (x,y,z,roll,yaw,pitch). Due to the absence of height information, the transformation relationship of the position is reduced from six degrees of freedom to three degrees of freedom (x,y,yaw). The final rotation matrix *R* and the translation matrix *T* can be represented as follows:

(5) $$R=\left[\begin{array}{cc} \cos(\theta_{y}) & -\sin(\theta_{y})\\ \sin(\theta_{y}) & \cos(\theta_{y}) \end{array}\right]\;T=\left[\begin{array}{c} t_{x}\\ t_{y} \end{array}\right]$$

These matrices are used to transform the coordinates from the infrastructure feature to the vehicle feature. The rotation matrix is used to rotate the coordinates, and the translation matrix is used to shift the coordinates.

In a manner similar to FFNet [42], we employed a compressor on the historical fusion features of the infrastructure $B_{inf}^{t-1,t}$ to mitigate the latency induced by communication transmission, eliminate superfluous information, and reduce transmission expenses. This compressor, which is composed of three Conv-Bn-ReLU blocks, reduces the feature size from [384,288,288] to a more manageable [12,36,36]. The compressed fused feature, along with its corresponding timestamps and calibration files, is then broadcast to the vehicle side. Upon receipt, the vehicle utilizes a decompressor, which is built from three Deconv-Bn-ReLU blocks, to restore the compressed fused features to their original size of [384,288,288].

### 3.3. Spatial Features Fusion Stage

In the CoFormerNet architecture, the transformer decoder layer on the infrastructure side follows the same structure as the LiDAR branch in the TransFusion [8] method. The details of this layer are not further elaborated on in this paper. The pre-detection boxes on the infrastructure side are transformed using the rotation and translation matrices described in Section 3.2. However, the hard association between the pre-detection boxes on the infrastructure-side and vehicle-side LiDARs can be affected by errors in sensor calibration and spatial mismatch caused by the continuous movement of the vehicle. To address this, CoFormerNet introduces a multi-head cross-attention mechanism to establish a soft association between the infrastructure-side LiDAR and the vehicle-side LiDAR. This allows the network to adaptively determine where and what information to obtain from the fused vehicle-side BEV features.

CoFormerNet tackles a fundamental hurdle in LiDAR-based object detection for cooperative perception: the spatial discrepancy between LiDAR data originating from the vehicle and infrastructure. This mismatch can mislead the network during the crucial cross-attention step, causing it to focus on irrelevant areas within the BEV feature map. This ultimately leads to inaccurate bounding box predictions and necessitates extended training times.

**Figure 4:**
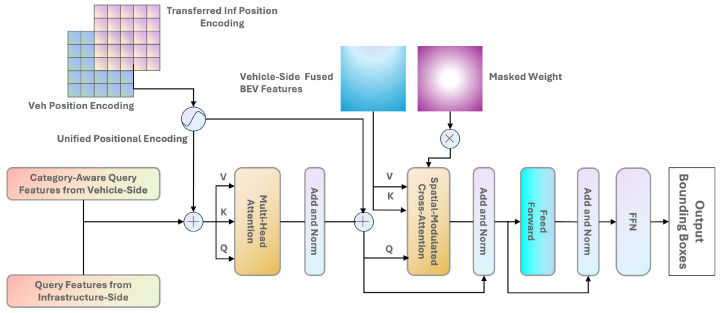
Architecture of the transformer decoder layer for vehicle-side fusion.

CoFormerNet addresses this issue in three ways. The detailed workings of this part are illustrated in Figure 4. First, instead of directly inputting the vehicle-side query features, we element-wise added the query features obtained from the infrastructure-side. This serves as useful auxiliary information when modeling the object context spatial relationship in the cross-attention module. Furthermore, during the prediction process, the infrastructure side, with its broader field of view, can provide valuable prior knowledge about objects, which is beneficial to target detection.

Second, traditionally, positional encodings for vehicle and infrastructure LiDAR data are generated independently, with different detection branches using their own positional encodings. Due to the different coordinate systems used by the infrastructure and vehicles, positional encoding is essential for feature-level fusion, ensuring consistency in feature representations from multiple sources. Since the input LiDAR BEV features already integrate historical infrastructure-side BEV features, we similarly transformed and integrated the infrastructure-side’s positional encodings. Borrowing from TransIFF [45], CoFormerNet transforms the infrastructure-side encoding to align with the vehicle’s coordinate system, enabling a unified encoding that allows for seamless information fusion and alignment across disparate spatial domains. This unified approach significantly enhances the network’s overall performance.

Finally, similar to Transfusion, we introduce the spatial-modulated cross-attention (SMCA) module [8]. Unlike Transfusion [8], which aims to reduce spatial feature fusion issues between different modalities using a cross-attention mechanism to bridge heterogeneity, in our scenario, the cross-attention inputs all come from LiDAR, addressing only spatial mismatches. SMCA uses 2D Gaussian masks centered on each object query within BEV features. These masks, similar to spotlights, guide the cross-attention process to prioritize relevant areas around the object, even if the sensor calibration is imperfect. The 2D Gaussian weight mask M is generated similarly to [46], using the formula $M_{ij}=\exp\left(-\frac{{\left(i-c_{x}\right)}^{2}+{\left(j-c_{y}\right)}^{2}}{\sigma r^{2}}\right)$, where (i,j) is the 2D center calculated by projecting the query prediction onto the image plane, r is the radius of the minimum circumscribed circle of the 3D bounding box projection angle, and σ is a hyper-parameter that adjusts the Gaussian distribution’s bandwidth. This weight map is then element-wise multiplied with the cross-attention maps of all attention heads. In this way, each object query focuses only on the relevant areas around the projected 2D box, enabling the network to better and more quickly learn the positions of image features based on the input LiDAR features.

By incorporating these innovative techniques, CoFormerNet effectively addresses the challenge of spatial inconsistency in LiDAR object detection. This not only improves the accuracy of the bounding box predictions but also reduces the training times, paving the way for more efficient and robust autonomous driving systems. After the SMCA module, another feed-forward network (FFN) is used to produce the final bounding box predictions using the object queries that contain both vehicle and infrastructure LiDAR information.

### 3.4. End-to-End Training

Previous methods often need to first train a certain branch for various reasons, and then retrain the entire network. This approach not only fails to fully utilize the data from both sides but also makes training more difficult and complex, making it impractical for real-world use. In FFNet [42], the 3D feature extraction backbone is first trained through vehicle–infrastructure data with zero latency, and then the feature flow is trained. The training objectives of the two training stages are not consistent, resulting in the first stage of training not being able to effectively improve the second stage. In the framework we designed, there is no need for this step-by-step training process. Instead, we can train the model in an end-to-end manner. This means that the entire model, from input to output, is trained all at once. This approach simplifies the training process and can often lead to better performance. In our case, end-to-end training allows the model to learn how to best fuse the data from different side sensors, handle communication delays, and learn how to make accurate object detection predictions. This holistic approach can lead to a more robust and accurate perception system for V2X perception.

## 4. Experiments

### 4.1. Experimental Settings

Our experimental verification was conducted on two datasets: DAIR-V2X [47] and V2XSet [48]. V2XSet was collected using simulations of CARLA [49] and OpenCDA [50], taking time delays into account. Before the release of the DAIR-V2X dataset, most algorithms were validated using simulated data, making it difficult to assess how these methods perform in real-world scenarios. However, the DAIR-V2X dataset was derived from the Beijing High-Level Autonomous Driving Demonstration Area, which covers 10 km of real urban roads, 10 km of highways, and 28 intersections. It includes 71,254 frames of image data and 71,254 frames of point cloud data from vehicle-mounted and roadside cameras, as well as vehicle-mounted and roadside LiDAR sensors. The dataset covers a wide range of conditions, including sunny, rainy, and foggy weather, as well as daytime and nighttime in urban and highway environments. Validation in the DAIR dataset better reflects the performance of the algorithms in real-world scenarios. Of course, we also provide a simple comparison with state-of-the-art algorithms on the V2XSet for reference.

During the feature extraction stage, we used the same structure for both the vehicle and infrastructure detection branches, employing VoxelNet [15] as the LiDAR backbone. To ensure fair comparisons between CoFormerNet and other methods, we used the same training and validation splits for all compared methods on both the DAIR-V2X and V2XSet datasets. All methods were evaluated using the same metrics [51], specifically BEV mAP and 3D mAP at IoU thresholds of 0.5 and 0.7. We reimplemented several baseline methods (e.g., VoxelNet [15], FFNet [42], TransIFF [45]) using the official codebases or provided implementations where available, ensuring consistent training and evaluation protocols. To simulate real-world scenarios, we introduced artificial latency during the evaluation phase and ensured that all methods were subjected to the same latency conditions for a fair comparison. All experiments were conducted on the same hardware setup (NVIDIA RTX 4090) to avoid discrepancies caused by different computational resources. We focused our evaluation on the car category, which also encompassed bus, truck, and van sub-classes. Finally, we limited the detection range for 3D objects to [0, −39.12, 100, 39.12], following the common practice in the field.

### 4.2. Results and Analysis

On the DAIR-V2X dataset, we compared CoFormerNet with four types of fusion methods: non-fusion (PointPillar [16] and VoxelNet [15]); and early fusion, late fusion, and intermediate fusion (FFNet [42] and TransIFF [45]). Additionally, we provided a camera-based fusion algorithm EMIFF [52] to compare the accuracy differences in 3D detection across different modalities.

Consistent with the FFNet [42] strategy, we tested the detection accuracy of various models with latencies of 200 and 300 ms. The comparison focused on two aspects: fusion methods and latency. For non-fusion, we used VoxelNet [15], which has a higher accuracy and efficiency compared with PointPillar [16] and AutoAlignV2 [53]. The results for non-fusion (PointPillars), early fusion, late fusion, and FFNet were obtained from the FFNet paper, and the results for EMIFF and TransIFF were obtained from their respective papers. We reimplemented VoxelNet [15] on the DAIR-V2X dataset for comparison purposes.

The results presented in Table 1 provide a comprehensive comparison of various fusion methods on the DAIR-V2X dataset, highlighting the superior performance of CoFormerNet over other methods. This analysis includes comparisons under different latency conditions to emphasize the robustness of CoFormerNet.

The performance of the camera-based method EMIFF was significantly lower compared with the LiDAR-based methods, illustrating the limitations of using camera-only data for accurate 3D detection. PointPillars and VoxelNet, which do not employ any fusion techniques, performed better than EMIFF. For example, VoxelNet achieved mAP@3D values of 52.40 at IoU 0.5 and 34.69 at IoU 0.7, with mAP@BEV values of 58.08 and 49.18. These LiDAR-based methods outperformed the camera-based EMIFF but still fell short of CoFormerNet, indicating the benefits of using LiDAR data. At the same time, these methods were less accurate compared with fusion methods, indicating that integrating information at the infrastructure level could effectively improve the detection accuracy.

Both early and late fusion methods showed a decline in performance with increased latency. Their results were lower than those of CoFormerNet, demonstrating CoFormerNet’s superior performance and robustness.

FFNet showed good performance but was affected by time delay. It still did not match CoFormerNet’s results, which were higher across all metrics. One significant reason why FFNet did not perform as well as CoFormerNet was that FFNet attempted to learn features corresponding to the current timestamp. However, due to the uncertainty of time delays, the features learned by FFNet often contained significant discrepancies. This time delay uncertainty introduced biases in the learned features, negatively impacting the model’s overall performance. CoFormerNet, on the other hand, does not directly address the problem of time delay. Instead, it transforms this challenge into a problem of effectively integrating historical information. By focusing on how to better fuse past data, CoFormerNet circumvents the issue of time delay uncertainty. This approach allows CoFormerNet to integrate both historical and current features more accurately, resulting in a more robust and reliable model. The emphasis on historical information fusion ensures that CoFormerNet can maintain high performance, even in the presence of temporal misalignments.

TransIFF performs well but focuses on instance-level feature fusion without considering the impact of time delay. This approach, while effective in a controlled environment, becomes impractical in real-world applications where time delays are inevitable. As a result, TransIFF’s performance significantly declines with increasing time delay, as evidenced by the experimental results. The lack of consideration for time delay introduces substantial inaccuracies in feature alignment, leading to lower overall performance compared with CoFormerNet, especially under latency conditions.

CoFormerNet’s minimal performance decline with increased latency demonstrates its robustness and reliability, making it suitable for real-world scenarios where latency is inevitable.

Our model achieved a new state-of-the-art level in the DAIR-V2X validation set, with a significant improvement over the previous best results. This indicates that our model can effectively utilize information from infrastructure sensors to improve the performance of autonomous driving systems.

On the V2XSet dataset, we compared CoFormerNet with two other intermediate fusion methods: V2X-ViT [48] and FFNet. The detailed results are shown in Table 2. CoFormerNet’s superior baseline performance in ideal conditions indicates its robust feature fusion strategy. The minimal decrease in CoFormerNet’s performance compared with the significant drops seen in V2X-ViT and FFNet underscores its effectiveness in handling time delays. CoFormerNet’s ability to maintain high performance even under significant latency conditions showcases its robustness and reliability in real-world scenarios where temporal misalignments are common.

### 4.3. Ablation Studies

To better understand the impact of each component of our model on the performance, we conducted a series of ablation studies. The results, presented in Table 3, indicate that each module, i.e., the temporal aggregation module (TAM), spatial-modulated cross-attention (SMCA), and end-to-end training, played a crucial role in enhancing the overall performance of our model.

The TAM effectively integrated temporal information from infrastructure sensors, which led to an improved detection accuracy. As shown in Table 3, incorporating the TAM improved the mAP@BEV (IoU = 0.5) by 8.85% under a latency of 200 ms compared with not using any module. Similarly, there was a 4.06% improvement with a 100 ms latency, demonstrating the significant impact of the TAM on performance. Introducing the SMCA at the spatial feature fusion stage could achieve performance levels comparable to zero-latency conditions, even under 200 ms latency. This highlights the module’s ability to mitigate the negative effects of spatial misalignment. End-to-end training allowed for the full exploitation and utilization of infrastructure data, simplifying and stabilizing the training process. Unlike FFNet, which requires two-step training, our model benefitted from a unified training process. This approach improved the performance by 1.6% compared with the non-end-to-end method, fully demonstrating the effectiveness of this training strategy. The ablation study clearly showed that the combination of all three modules (TAM, SMCA, and end-to-end training) produced the highest performance across both the mAP@3D and mAP@BEV metrics.

The introduction of the TAM significantly increased the integration of temporal information, which led to substantial improvements in the detection accuracy. SMCA effectively addressed the spatial feature fusion, which mitigated the adverse effects of spatial asynchrony. End-to-end training optimized the use of vehicle and infrastructure data, which enhanced the overall stability and performance of the model. These findings underscore the importance of each component in our model and their collective contribution to achieving a state-of-the-art performance in VIC3D object detection tasks.

To validate the practicality of our proposed method in real-world scenarios, we compared the computational costs of several methods. Although the methods are not fully real-time yet, advancements in computational hardware are expected to bridge this gap. The speed tests were conducted on an NVIDIA RTX 4090. Table 4 shows a clear trade-off between the computational efficiency and detection accuracy among the different models. CoFormerNet, while having the longest inference time, delivered the highest detection accuracy. This demonstrates its effectiveness but also highlights the need for further optimization to achieve real-time performance. With advancements in computational hardware, the gap to real-time performance is expected to close, making CoFormerNet a viable option for high-accuracy 3D object detection in real-world scenarios.

**Figure 5:**
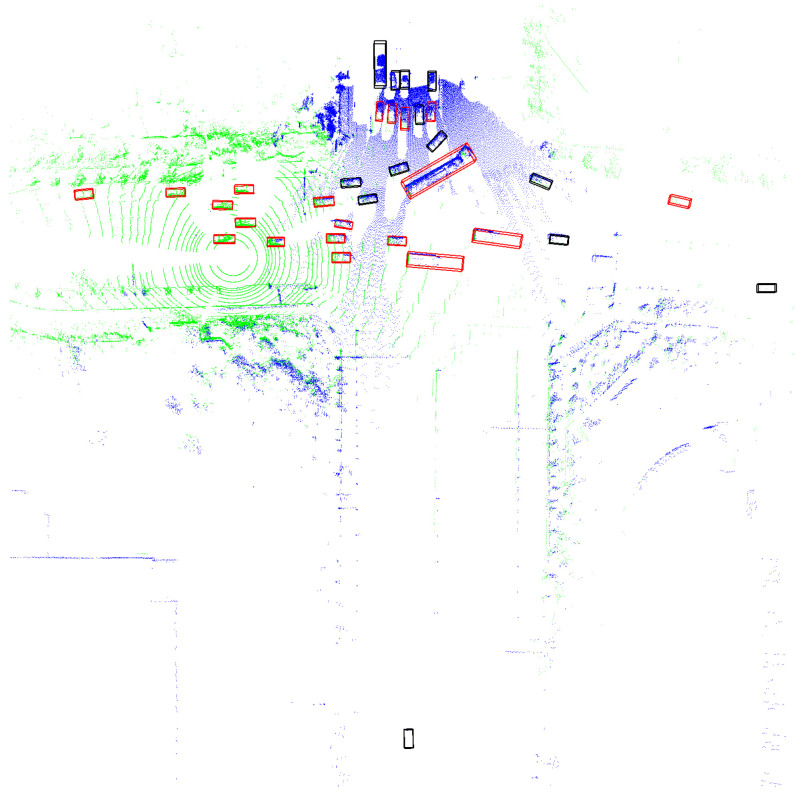
The detection results, where the blue point clouds represent data from infrastructure sensors, the green point clouds represent data from vehicle sensors, the red boxes indicate detections from individual vehicles, and the black boxes highlight additional detections obtained through sensor fusion. The fields of view of the vehicle and infrastructure sensors overlapped.

**Figure 6:**
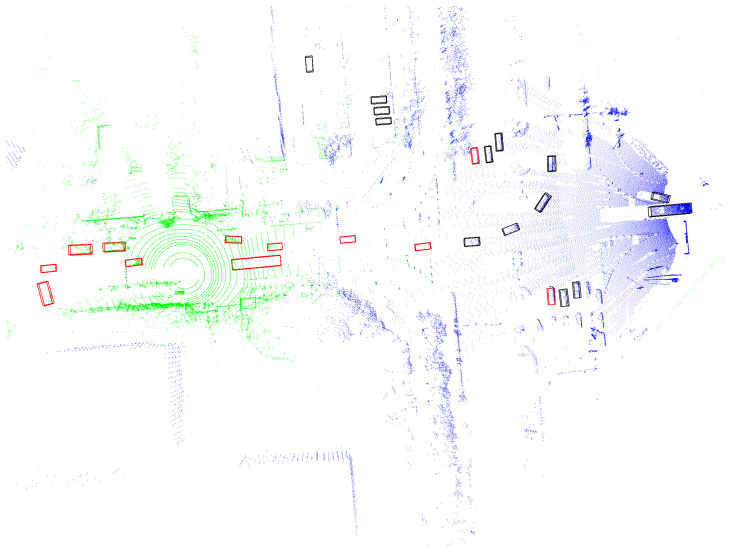
The detection results, where the blue point clouds represent data from infrastructure sensors, the green point clouds represent data from vehicle sensors, the red boxes indicate detections from individual vehicles, and the black boxes highlight additional detections obtained through sensor fusion. The fields of view were directly facing each other.

Finally, to provide a more intuitive understanding of the role our method plays in collaborative perception, Figure 5 and Figure 6 present detection results in two common scenarios. In Figure 5, the fields of view of the vehicle and infrastructure sensors overlap, while in Figure 6, the fields of view are directly facing each other. In these scenarios, the blue point clouds represent data captured from infrastructure sensors, and the green point clouds represent data captured from vehicle sensors. The red boxes indicate detections from individual vehicles, and the black boxes highlight additional detections obtained through sensor fusion. The overlapping fields of view between the vehicle and infrastructure sensors enhance the detection accuracy by providing complementary perspectives, leading to more comprehensive and accurate object identification. The detailed detection results in these scenarios underscore the practical benefits of sensor fusion, highlighting the potential for improved safety and performance in real-world applications.

## 5. Conclusions

CoFormerNet is a novel cooperative detection framework designed to address the vehicle–infrastructure cooperative 3D (VIC3D) object detection problem, with the aim of improving safety and performance in challenging traffic scenarios for autonomous driving systems. This framework capitalizes on the advantages of infrastructure sensors while overcoming the temporal asynchrony limitations caused by communication latency in VIC3D object detection. The key contributions of CoFormerNet include spatio-temporal cross-domain feature fusion, end-to-end training, and state-of-the-art performance. These contributions demonstrate that CoFormerNet is an effective and practical VIC3D object detection framework with broad application prospects.

## Figures and Tables

**Table 1:** Comparison of different fusion methods on the DAIR-V2X datset. CoformerNet significantly outperformed all other fusion methods. * indicates that EMIFF is a camera-based intermediate fusion method. - indicates that the original paper does not provide the corresponding metrics.

Model	Fusion Type	Latency	mAP@3D		mAP@BEV	
			IoU = 0.5	IoU = 0.7	IoU = 0.5	IoU = 0.7
EMIFF *	Intermediate	0	15.61	-	21.44	-
PointPillars	Non-fusion	/	48.06	-	52.24	-
VoxelNet	Non-fusion	/	52.40	34.69	58.08	49.18
FFNet	Intermediate	0	55.81	30.23	63.54	54.16
TransIFF	Intermediate	0	59.62	46.03	-	-
CoFormerNet	Intermediate	0	61.03	39.14	69.33 (+11.7)	54.59
Early Fusion	Early	200	54.63	38.23	61.08	50.06
Late Fusion	Late	200	52.43	36.54	58.10	49.25
FFNet	Intermediate	200	55.37	31.66	63.20	54.69
TransIFF	Intermediate	200	53.47	37.21	-	-
CoFormerNet	Intermediate	200	60.97	38.97	69.13 (+11.5)	54.65
Early Fusion	Early	300	51.37	37.25	58.28	49.81
Late Fusion	Late	300	51.35	36.24	56.89	48.79
FFNet	Intermediate	300	53.46	30.42	61.20	52.44
TransIFF	Intermediate	300	51.02	31.74	-	-
CoFormerNet	Intermediate	300	60.63	37.28	68.60 (+10.52)	53.29

**Table 2:** Comparison of different fusion methods on V2XSet datset. CoFormerNet also significantly outperformed all other fusion methods.

Model	Fusion Type	Latency	mAP@3D	
			IoU = 0.5	IoU = 0.7
V2X-ViT	Intermediate	0	88.23	71.27
FFNet	Intermediate	0	89.47	72.66
CoFormerNet	Intermediate	0	90.23	72.95
V2X-ViT	Intermediate	200	83.61	61.49
FFNet	Intermediate	200	85.45	70.23
CoFormerNet	Intermediate	200	89.28	71.02
V2X-ViT	Intermediate	300	80.71	54.66
FFNet	Intermediate	300	83.31	57.93
CoFormerNet	Intermediate	300	84.22	59.01

**Table 3:** The ablation study results of different modules (TAM, SMCA, and End2End). The results indicate that the combination of all three modules yielded the highest performance across both metrics.

TAM	SMCA	End2End	Latency	mAP@3D		mAP@BEV	
				IoU = 0.5	IoU = 0.7	IoU = 0.5	IoU = 0.7
×	×	×	0	55.67	35.12	63.78	54.26
✓	×	×	0	58.25	36.27	67.84	54.31
✓	✓	✓	0	60.67	39.12	70.78	55.26
×	×	×	200	52.40	34.69	58.08	49.48
×	✓	×	200	55.40	34.44	63.14	52.32
✓	×	×	200	57.11	35.09	66.93	52.87
✓	✓	×	200	59.24	36.06	67.53	54.02
✓	✓	✓	200	60.97	38.97	69.13	54.65

**Table 4:** Performance comparison of different models.

Model	Time	mAP@3D (IoU = 0.5)
PointPillars	31 ms	48.06
VoxelNet	95 ms	52.40
TransIFF	110 ms	59.62
FFNet	101 ms	55.81
CoFormerNet	122 ms	61.03

## Data Availability

Not applicable.
